# Supplementary material for: A Discretized Overlap Resolution Algorithm (DORA) for resolving spatial overlaps in individual-based models of microbes
Source: PLoS Comput Biol. 2025 Apr 21;21(4):e1012974. doi: 10.1371/journal.pcbi.1012974 (PMC12124742; doi:10.1371/journal.pcbi.1012974)
Supplement: S1 Algorithm — (PDF) [file pcbi.1012974.s001.pdf]

S1 Algorithm. Algorithm Implementation Using the Moore Neighborhood

Forward Translation

For each grid unit (i, j), the occupancy of a cell k is calculated as:

$$\Omega_{ij}^{(k)} = \frac{\max(0, \min(i+1, x_{\text{right}}) - \max(i, x_{\text{left}})) \times \max(0, \min(j+1, y_{\text{top}}) - \max(j, y_{\text{bottom}}))}{w \times h}$$

The total occupancy for each grid unit (i, j) is given by:

$$\Omega[i][j] = \sum_k \Omega_{ij}^{(k)}$$

Overlap Resolution

Excess occupancy for each grid unit (i, j) is calculated as:

$$E_{ij} = \max(0, \Omega_{ij} - 1)$$

For the Moore neighborhood, the sum of excess occupancies from all 8 neighboring cells is given by:

$$E_{i,j}^{\mathcal{M}} = \sum_{(k,l) \in \mathcal{M}_{ij}} E_{kl}$$

The updated occupancy matrix Ω' is then computed as:

$$\Omega'_{ij} = \Omega_{ij} - \alpha \cdot \left(E_{ij} - \frac{1}{8} \cdot E_{i,j}^{\mathcal{M}} \right)$$

Back-Translation and Movement

For each grid unit (i, j), the local movement vector $(v_{i,j}^x, v_{i,j}^y)$ is calculated based on the displacements recorded in the motion matrix M:

$$v_{i,j}^x = (M_{ij}^{(0)} - M_{ij}^{(1)}) + \frac{1}{\sqrt{2}} \cdot (M_{ij}^{(4)} - M_{ij}^{(5)} + M_{ij}^{(6)} - M_{ij}^{(7)})$$

$$v_{i,j}^y = (M_{ij}^{(2)} - M_{ij}^{(3)}) + \frac{1}{\sqrt{2}} \cdot (M_{ij}^{(4)} - M_{ij}^{(6)} + M_{ij}^{(5)} - M_{ij}^{(7)})$$

Here, $M_{ij}^{(0)}$ corresponds to rightward motion, $M_{ij}^{(1)}$ corresponds to leftward motion, $M_{ij}^{(2)}$ corresponds to upward motion, $M_{ij}^{(3)}$ corresponds to downward motion, and $M_{ij}^{(4)}$, $M_{ij}^{(5)}$, $M_{ij}^{(6)}$, and $M_{ij}^{(7)}$ represent the diagonal motions (top-right, top-left, bottom-right, bottom-left).

For cells spanning multiple grid units, the total movement vector (V^x, V^y) is determined by summing the effects from all grid units occupied by the cell:

$$V^x = \sum_{(i,j) \in \text{Cell's grid units}} v_{i,j}^x, \quad V^y = \sum_{(i,j) \in \text{Cell's grid units}} v_{i,j}^y$$
