# Supplementary material for: A Discretized Overlap Resolution Algorithm (DORA) for resolving spatial overlaps in individual-based models of microbes
Source: PLoS Comput Biol. 2025 Apr 21;21(4):e1012974. doi: 10.1371/journal.pcbi.1012974 (PMC12124742; doi:10.1371/journal.pcbi.1012974)
Supplement: S1 Table — (PDF) [file pcbi.1012974.s002.pdf]

S1 Table. Parameter Values Used in Simulations

The nominal values of all parameters used in the simulations can be found in Table 1.

**Parameter**	**Interpretation**	**Units**	**Value**
μ_{max}	Maximum growth rate	min^{-1}	0.0176
K_{N}	Monod half-saturation constant	$\mathrm{g\ l}^{-1}$	0.001078
Y_{max}	Apparent yield at μ_{max}, corrected for maintenance	gDW/g nutrient	0.45
$V_{\mathrm{d,min}}$	Minimum volume at division	l	2.6×10^{-15}
D_{i}	Diffusion coefficient of substance i	$\mu\mathrm{m}^2\mathrm{min}^{-1}$	815
ρ	Density of cellular biomass	$\mathrm{gDW\ l}^{-1}$	290.0
k	Intercellular distance factor in cell shoving mechanism	NA	1.3
CV	Coefficient of variance of specific growth rate	NA	0.1
α	Numerical diffusivity coefficient	NA	0.05

Table 1: Parameter Values and Definitions [1, 2].

References

[1] Kreft, J.U., 2004. Biofilms promote altruism. *Microbiology* 150, 2751–2760.

[2] Tack, I.L., Logist, F., Fernández, E.N., Van Impe, J.F., 2015. An individual-based modeling approach to simulate the effects of cellular nutrient competition on *Escherichia coli* K-12 MG1655 colony behavior and interactions in aerobic structured food systems. *Food Microbiology* 45, 179–188.
