# Supplementary material for: A Discretized Overlap Resolution Algorithm (DORA) for resolving spatial overlaps in individual-based models of microbes
Source: PLoS Comput Biol. 2025 Apr 21;21(4):e1012974. doi: 10.1371/journal.pcbi.1012974 (PMC12124742; doi:10.1371/journal.pcbi.1012974)
Supplement: S2 Algorithm — (PDF) [file pcbi.1012974.s003.pdf]

S2 Algorithm. Algorithm Implementation with Stochastic Motion

This subsection presents the modifications made to the original Discretized Overlap Resolution Algorithm (DORA) to incorporate stochastic motion. The stochastic version introduces random displacements to all cells at each iteration, simulating the variability often observed in biological systems. This addition has a computational complexity of $O(N)$, resulting in negligible overhead to the overall algorithm's efficiency. Stochastic displacement may be particularly useful for simulating microbial systems where random motion is biologically relevant, such as in motile cells or environments with inherent variability due to fluctuating external conditions, enhancing the biological realism of the simulations. Below, we describe the changes to the algorithm:

Stochastic DORA Algorithm Overview

The primary modification to the original DORA algorithm is the addition of a stochastic motion step before the overlap resolution phase. At each iteration, all cells are displaced by a small random vector sampled from a Gaussian distribution with a standard deviation σ. The updated algorithm is outlined as follows:

Algorithm 1 Stochastic Discretized Overlap Resolution Algorithm (Stochastic DORA)

```
Input: Spatial attributes of cells (positions, radii), random displacement standard deviation σ
Output: Adjusted positions of cells to resolve overlaps
Apply Stochastic Motion
for each cell do
    Generate a random displacement vector (Δx, Δy) ~ 𝒩(0, σ^2)
    Update cell position based on (Δx, Δy), ensuring boundary conditions are respected
end for
Forward Translation
for each cell do
    Compute cell boundaries relative to grid units
    Update occupancy matrix Ω based on cell's spatial extent
end for
Overlap Resolution
Initialize excess matrix E from Ω
while excess occupancy exists do
    for each grid unit (i, j) do
        Compute excess occupancy E_ij
        Redistribute excess to adjacent units, update Ω
        Update motion matrix M based on excess redistribution
    end for
end while
Backward Translation
for each cell do
    Compute movement vector from M
    Adjust cell position based on movement vector
end for
```

The performance of the stochastic DORA algorithm was evaluated through simulations conducted under identical parameters with varying stochastic realizations. The results demonstrate the algorithm's robustness in maintaining low overlap ratios and its ability to generate diverse spatial configurations. Figure 1 shows the evolution of the overlap ratio over time, while Figure 2 highlights the spatial variability observed across six independent stochastic runs. Simulations were performed for colony growth under a scarce nutrient concentration of 1 mg/L, with a random displacement vector standard deviation of 0.01 μm.

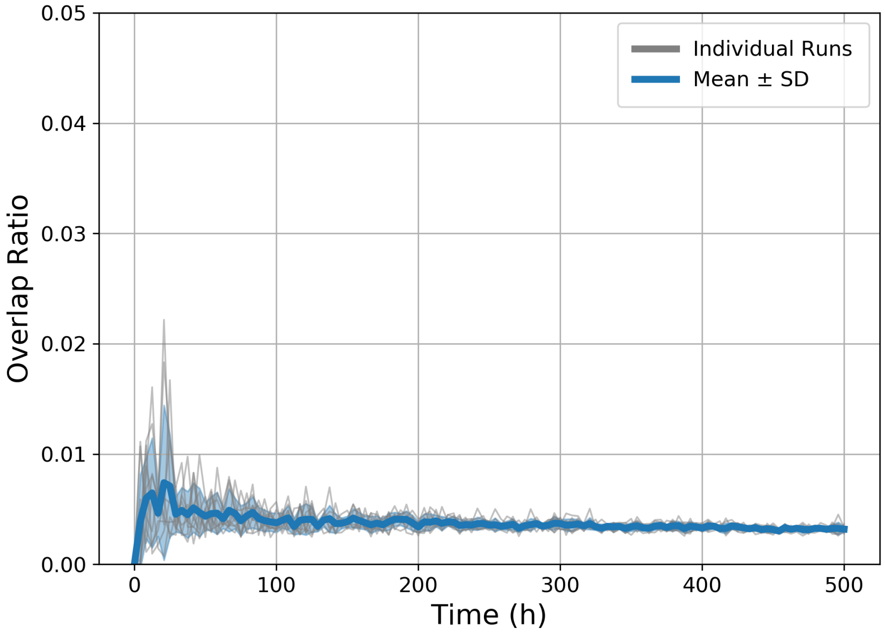

Figure 1: The evolution of the overlap ratio over time when using the stochastic DORA algorithm. The figure shows the mean and standard deviation (blue line with shaded region) of the overlap ratio across ten independent runs, as well as the individual runs (gray lines). Simulations were conducted under a scarce nutrient concentration of 1 mg/L, with a random displacement vector standard deviation of 0.01 μm. The results demonstrate the robustness of the stochastic DORA algorithm in maintaining low overlap ratios despite the variability introduced by stochastic displacement.

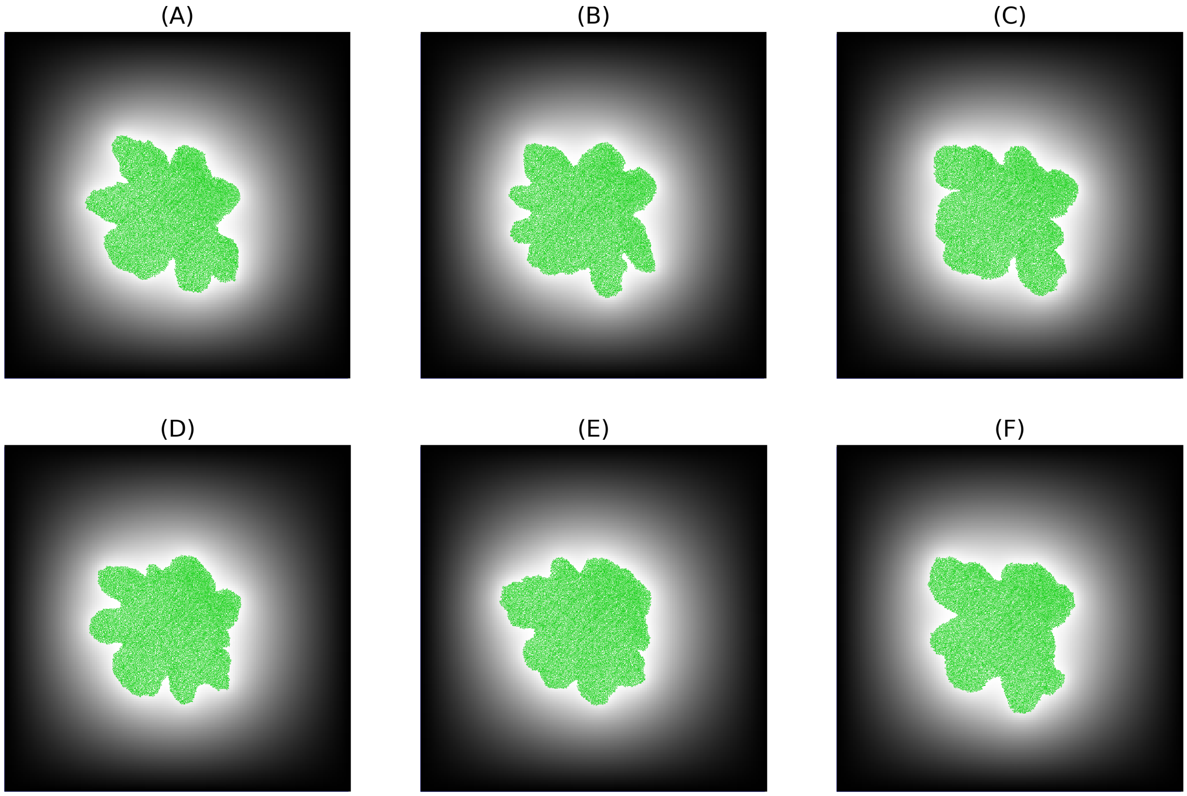

Figure 2: Final states of six simulation runs using the stochastic DORA algorithm. Simulations were conducted under a scarce nutrient concentration of 1 mg/L, with snapshots taken at the end of the simulation (500 hours). The random displacement vector had a standard deviation of 0.01 μm. Subplots (A), (B), (C), (D), (E), and (F) represent six unique stochastic realizations under identical parameters, illustrating the spatial variability introduced solely by stochastic motion.
