# Supplementary material for: A Discretized Overlap Resolution Algorithm (DORA) for resolving spatial overlaps in individual-based models of microbes
Source: PLoS Comput Biol. 2025 Apr 21;21(4):e1012974. doi: 10.1371/journal.pcbi.1012974 (PMC12124742; doi:10.1371/journal.pcbi.1012974)
Supplement: Attachment — Submitted filename: reviewing_PlosCB_Hashem.pdf [file pcbi.1012974.s005.pdf]

The manuscript “A Discretized Overlap Resolution Algorithm (DORA) for Resolving Spatial Overlaps in Individual-based Models of Microbes” by Hashem, Wang, and Van Impe presents a novel algorithm, DORA, to reduce the computational time and memory resources needed in individual-based models (IbMs) of growing cells in a 2D space. In particular, DORA resolves the cell-to-cell overlapping occurring at each step of IbM simulations. Their idea is to translate the spatial distribution of cells to an occupancy grid, which indicates if a given square in the grid is occupied by more than two cells. If a square is occupied by more than one cell, the next stage of DORA is to iteratively identify the movements necessary to alleviate the overlaps and then execute those movements. The overlaps can not be entirely removed as DORA is an approximation, but they are below 1% for the simulations shown. The resulting computing time is less than for exact algorithms, such as arrays or kd-trees, especially when the number of cells in a simulation is large (e.g. exceeding 10^4 cells).

The article tackles a relevant problem for IbMs, and offers a promising simple solution to enable larger and faster simulations of cell cultures. Given the importance of communicating the algorithm, most of the text clearly explains it and benchmarks it against kd-trees to resolve cell overlaps. The paper is of general interest and likely to be a valuable contribution to Plos Computational Biology worthy of publication. The idea motivating the algorithm is sound and intuitive, however, I would like to see certain clarifications. I provide major and minor comments/suggestions, in no particular order.

Major:

1. For replicability:

2.1 Please make code and data accessible.

2.2 Please provide the Monod parameters, and others, used in your simulations.

2. The explanation of the algorithm in Figure 1 and its preceding text is clear, similar to the *Algorithm 1* pseudocode. However, the explanation of the sections *Forward Translations*, *Overlap Resolution*, and *Backward Translation and Movement* seems overly complicated. In particular:

2.1 Having the *Algorithm 1* pseudocode before these sections would ease understanding, as it gives intuition and the connections between the three stages of DORA.

2.2 Having a figure to exemplify the geometric aspect of the equations would be very useful in understanding them.

2.3 It might help to explain the derivations of equations, including 1) that you approximate cells as “squares” (as you state in the Results). 2) Please mention any other approximations being made. 3) Where does the ¼ term come from in all the equations? 3) It

would be useful to say what are the neighboring cells in a Von Neumman neighborhood. 4) You could say that E_{ij} - E_{ij}^{N} is a gradient, and 5) write $M_{i,j}^{(bottom)}$ instead of $M_{i,j}^{(0)}$, etc, for clarity.

2.4 The units of the equations for $x_{left/right}$ and $y_{bottom/top}$ seem to be incorrect. If I understand correctly x_c, y_c, r, w, and h have units of length. Thus, how can the ratios r/w and r/h be added to x_c and y_c? I might be understanding this wrong.

2.5 It would be useful to explain how you choose appropriate w and h for the squares.

2.6 Equations could be numbered to refer to them.

3. There are multiple instances in *Results and Discussion*, where citations are needed, e.g. about 1) the expected colony edges obtained for high, medium, and low nutrients, 2) the features of the k-d tree methods, and 3) the link between overlap resolution and PDEs, etc.

4. It seems necessary to specify throughout the text that the cell shape is a circle. Or, is it possible to simulate other cell shapes?

Minor:

1. The abstract could benefit from mentioning 1) how IbMs differ from cellular automata, 2) that IbMs already consider space as being discrete, and 3) the conventional methods to solve overlaps (i.e. arrays and kd-trees).

2. Figure 1 could be modified to explain how arrays or kd-trees are constructed. Such modifications could be either in the graphic or caption.

3. An intermediate peak is observed in the *Overlap Ratio* of Figure 4(d) – around 5 to 10 hours. Have the authors investigated the reason?

4. Please signal in Figure 3(a) the cell overlaps that you mention in the text.

5. Please consider putting the names *DORA* or *kd-tree* on top of the panels where growth simulations are shown (Figures 3 to 8).

6. It would be better to reduce the range of the y-axis in figures showing the *Overlap Ratio* (Figures 3 to 8).

7. In figures showing the *Resolution Time* (Figures 3 to 8), you write $X10^3$ *ms*. But, is not this just seconds (*s*)? Please consider rewriting as seconds.

8. Please remove the first paragraph in *Results and Discussion* which is a loose sentence (a copy from within paragraph 2).

9. In Materials and Methods, first paragraph, Figure 3(a) is cited instead of Figure 1(a).

10. Please check typos, e.g. *Inidividual*, *to produce the replicate*, *g/L*, etc.
