# Supplementary material for: A Discretized Overlap Resolution Algorithm (DORA) for resolving spatial overlaps in individual-based models of microbes
Source: PLoS Comput Biol. 2025 Apr 21;21(4):e1012974. doi: 10.1371/journal.pcbi.1012974 (PMC12124742; doi:10.1371/journal.pcbi.1012974)
Supplement: Attachment — Submitted filename: ReplyToReviews.pdf [file pcbi.1012974.s006.pdf]

Replies to the Reviewers' Comments

We would like to thank the editor and the reviewers for their valuable comments and suggestions. We believe the revisions have significantly improved the manuscript. Below, we provide responses to each comment and describe the changes made in the manuscript. Changes in the manuscript are highlighted in red.

Replies to Reviewer 1:

The manuscript introduces the Discretized Overlap Resolution Algorithm (DORA) aimed at resolving cellular overlaps in individual-based models (IBMs) of microbial growth. DORA uses a grid-based framework to manage spatial overlaps, reducing computational costs compared to traditional pairwise comparison methods. The authors evaluate DORA's performance in simulating microbial colonies and biofilms under different nutrient conditions, demonstrating its computational efficiency and accuracy.

Comment 1.1: The manuscript primarily focuses on a deterministic approach. Consideration of stochastic elements, which are often relevant in microbial modeling, would broaden the applicability of the algorithm. How would the algorithm adapt to stochastic IBMs, where random elements influence cell behavior and interactions?

Reply 1.1: We appreciate the reviewer's suggestion to consider stochastic elements. While DORA operates deterministically, it can be integrated into a stochastic IbM framework to capture biological variability. Additionally, DORA can be adapted to incorporate stochastic movements by introducing a random motion vector before resolving overlaps.

The following text has been added to the Conclusion section, lines 395-406:

DORA, in contrast, operates as a deterministic algorithm. It systematically resolves spatial overlaps based on a fixed set of rules within a discretized grid framework. However, DORA is designed to function within a broader IbM framework, which often incorporates stochastic elements to simulate biological variability. For example, random components can be introduced to capture variability between individual cells in processes such as growth, replication, and death. This approach allows for a hybrid model where spatial resolution is handled deterministically, while biological behaviors are influenced by stochastic variability. Furthermore, it is possible to adapt DORA to incorporate stochastic movements by applying a stochastic motion vector to cells before executing the overlap resolution process. This adaptation could introduce randomness to the movement patterns, allowing for simulations that more accurately reflect the inherent stochasticity observed in real biological systems.

Comment 1.2: The algorithm handles horizontal and vertical movements. Diagonal movements should also be considered to fully capture the spatial dynamics of microbial growth.

Reply 1.2: We agree with the reviewer, and in fact, when we initially developed the algorithm, we considered both a Von Neumann neighborhood and a Moore neighborhood, which includes diagonal neighbors. However, when testing the Von Neumann neighborhood, the results for colony growth demonstrated uniform, nearly circular growth with no bias toward the cardinal directions. This is likely due to the stochastic elements averaging out any directional biases. Furthermore,

testing with the Moore neighborhood showed no significant difference. As a result, we opted to proceed with the simpler algorithm.

We have added the Moore neighborhood implementation of the algorithm to the supplementary files, and the following paragraph has been added to lines 260-269:

In these simulations, we utilized a Von Neumann neighborhood to handle overlap resolution. While this neighborhood is anisotropic, as it emphasizes movements along the cardinal directions and underrepresents diagonal movements, the colonies still exhibited nearly circular growth. This suggests that the spatial dynamics of colony expansion were not strongly impacted by the anisotropy of the overlap resolution step. We attribute this to the stochastic elements inherent in the simulation, such as random cell reproduction and placement, which likely averaged out any directional biases introduced by the choice of neighborhood. Additionally, we tested an alternative configuration of the algorithm using a Moore neighborhood (see supplementary material) but found no significant difference in the observed growth patterns or overlap ratios.

Comment 1.3: The manuscript lacks a comparative analysis with other off-lattice agent-based modeling techniques or the particle Lenia approach. These methods allow for arbitrary ODEs to adjust particle overlap, potentially offering more flexibility. Highlighting the advantages and limitations of DORA relative to these approaches would strengthen the manuscript. A detailed discussion of the superiorities of DORA compared to other existing methods (other than kd-tree) would be beneficial. This includes scenarios where DORA excels and where it might be limited.

Reply 1.3: Following the reviewer's suggestion, we have incorporated a comparative analysis of DORA with off-lattice methods like particle Lenia, highlighting the strengths and limitations of both approaches.

The Conclusion section has been expanded with the following paragraphs, lines 383-415:

While we have demonstrated DORA's advantages over a kd-tree-based approach, its performance relative to other off-lattice agent-based modeling techniques, such as the particle Lenia approach (Chan (2018)), provides an interesting contrast. Unlike DORA, which operates in a discretized space with deterministic overlap resolution, the particle Lenia approach employs a continuous spatial framework that allows for the flexible application of ordinary differential equations (ODEs) to model interactions and resolve overlaps between particles. This continuous framework facilitates the incorporation of stochastic elements, making it particularly effective for simulating systems where randomness plays a critical role, such as microbial motility, diffusion processes, and environmental fluctuations. Moreover, the ability to dynamically adjust particle interactions via ODEs affords particle Lenia a high degree of flexibility in capturing emergent behaviors driven by non-linear dynamics and stochasticity (Horibe et al. (2023), Jain et al. (2023)).

DORA, in contrast, operates as a deterministic algorithm. It systematically resolves spatial overlaps based on a fixed set of rules within a discretized grid framework. However, DORA is designed to function within a broader IbM framework, which often incorporates stochastic elements to simulate biological variability. For example, random components can be introduced to capture variability between individual cells in processes such as growth, replication, and death. This approach allows for a hybrid model where spatial resolution is handled deterministically, while biological behaviors are influenced by stochastic variability. Furthermore, it is possible to adapt DORA to incorporate stochastic movements by applying a stochastic motion vector to cells before executing the overlap resolution process. This adaptation could introduce randomness to the movement patterns, allowing for simulations that more accurately reflect the inherent stochasticity observed in real biological systems.

Despite the flexibility and computational efficiency of particle Lenia, significant challenges remain in applying it to biological systems. A key difficulty lies in translating complex biological phenomena into the parameters required by particle Lenia. Formulating ODEs that faithfully represent the dynamics of biological interactions demands a deep understanding of the underlying mechanisms and requires fine calibration of these equations. While continuous, flexible parameter spaces in particle Lenia offer great versatility, they may oversimplify biological processes, poten-

tially obscuring mechanistic understanding. This becomes particularly problematic when specific biological interactions or constraints need to be explicitly represented (Wu and Voit (2009), Ji et al. (2017)).

Comment 1.4: The description of the algorithm is clear but could benefit from more illustrative examples or pseudocode to guide readers through the steps, especially those less familiar with computational modeling.

Reply 1.4: An additional figure (Figure 3) has been added to visually illustrate the steps of the algorithm for better clarity.

Comment 1.5: On page 2, the manuscript should refer to Figure 1, not Figure 3. Please ensure accurate figure references.

Reply 1.5: Corrected.

Comment 1.6: Ensure consistent use of terminology throughout the manuscript. For example, the terms "cell" and "bacterial cell" are used interchangeably; choose one term for consistency.

Reply 1.6: The manuscript has been proofread, and terminology has been made consistent throughout. Thank you for the constructive feedback.

Replies to Reviewer 2:

The article tackles a relevant problem for IbMs, and offers a promising, simple solution to enable larger and faster simulations of cell cultures. Given the importance of communicating the algorithm, most of the text clearly explains it and benchmarks it against kd-trees to resolve cell overlaps. The paper is of general interest and likely to be a valuable contribution to PLOS Computational Biology, worthy of publication. The idea motivating the algorithm is sound and intuitive. However, I would like to see certain clarifications. I provide major and minor comments/suggestions, in no particular order.

Comment 2.1: For replicability: 1) Please make code and data accessible. 2) Please provide the Monod parameters, and others, used in your simulations.

Reply 2.1: The code and data have been deposited to an online repository, and the detailed biological parameters for the simulations have been added as a supplementary file.

Comment 2.2: The explanation of the algorithm in Figure 1 and its preceding text is clear, similar to the Algorithm 1 pseudocode. However, the explanation of the sections Forward Translations, Overlap Resolution, and Backward Translation and Movement seems overly complicated.
Sub-comment 2.2.1: Having the Algorithm 1 pseudocode before these sections would ease understanding, as it gives intuition and the connections between the three stages of DORA.

Reply 2.2.1: The pseudocode has been displaced to be before these sections.

Sub-comment 2.2.2: Having a figure to exemplify the geometric aspect of the equations would be very useful in understanding them.

Reply 2.2.2: Figure 3 has been added to help illustrate the equations.

Sub-comment 2.2.3: It might help to explain the derivations of equations, including: 1) The

approximation of cells as "squares" (as you state in the Results). 2) Mentioning any other approximations being made. 3) Explaining where the $\frac{1}{4}$ term comes from in the equations. 4) Clarifying what neighboring cells are in a Von Neumann neighborhood. 5) Using clearer notation like $M_{i,j(\text{bottom})}$ instead of $M_{i,j(0)}$.

Reply 2.2.3: The assumptions of the model have been clarified. New text has been added to explain the Von Neumann neighborhood and the origin of the 1/4 term, with notation properly adjusted.

The following text has been added, lines 207-216:

Several approximations are employed to manage the computational complexity of the algorithm. Specifically, (i) circular cells are approximated as squares to simplify boundary calculations and overlap detection. In addition, (ii) the simulation environment is discretized into a grid, which limits spatial precision as the fixed grid resolution may not capture finer-scale details. Furthermore, (iii) cells are treated as rigid, non-deformable entities, an assumption also utilized in the kd-tree-based approach to facilitate the overlap resolution process. While this method effectively resolves overlaps for circular cells, it does not account for rotational forces. As a result, simulating cells with more complex shapes, such as rod-shaped cells, would require additional modifications, which are not considered in the current framework.

and lines 174-176:

The $\frac{1}{4}$ term arises from the Von Neumann neighborhood, where each grid unit has four neighboring cells (up, down, left, and right). This ensures that excess occupancy is redistributed evenly across the four adjacent cells.

and lines 166-168:

The term $E_{ij} - E_{i,j}^{\mathcal{N}}$ represents a local gradient of excess occupancy, driving the redistribution of cells to neighboring grid units. The diffusion process and the redistribution of excess occupancy are shown in Figure 3(b).

Comment 2.2.4: The units of the equations for $x_{\text{left/right}}$ and $y_{\text{bottom/top}}$ seem to be incorrect. Please explain how the ratios r/w and r/h can be added to x_c and y_c.

Reply 2.2.4: We agree that this can be confusing. A clarifying text has been added to explain the units, lines 130-132:

Here, x_c and y_c are the coordinates of the cell's center in grid units, r is the cell's radius in μm, and w and h represent the width and height of a grid unit in μm per grid unit.

Comment 2.2.5: It would be useful to explain how you choose appropriate w and h for the squares.

Reply 2.2.5: The scale of the grid unit has been clarified. New text added, lines 132-133:

The values of w and h are chosen to be on the same scale as the cell radius, typically around 1 μm per grid unit.

Comment 2.2.6: Equations could be numbered to refer to them.

Reply 2.2.6: Equations have been numbered accordingly.

Comment 2.3: There are multiple instances in Results and Discussion where citations are needed, e.g., about: 1) The expected colony edges for high, medium, and low nutrients. 2) The features of kd-tree methods. 3) The link between overlap resolution and PDEs.

Reply 2.3: New citations have been added.

Comment 2.4: It seems necessary to specify throughout the text that the cell shape is a circle. Or, is it possible to simulate other cell shapes?

Reply 2.4: This is correct, text has been added to clarify the current limitations, lines 214-217:

While this method effectively resolves overlaps for circular cells, it does not account for rotational forces. As a result, simulating cells with more complex shapes, such as rod-shaped cells, would require additional modifications, which are not considered in the current framework.

Minor Comments:

Comment 2.5: The abstract could benefit from mentioning: 1) How IbMs differ from cellular automata. 2) That IbMs already consider space as being discrete. 3) The conventional methods to solve overlaps (e.g., arrays and kd-trees).

Reply 2.5: The abstract has been adjusted accordingly.

Comment 2.6: Figure 1 could be modified to explain how arrays or kd-trees are constructed. Such modifications could be either in the graphic or caption.

Reply 2.6: The caption has been edited to explain how arrays and kd-trees are constructed.

Comment 2.7: An intermediate peak is observed in the Overlap Ratio of Figure 4(d) â€“ around 5 to 10 hours. Have the authors investigated the reason?

Reply 2.7: An explanation has been added, lines 332-337:

Notably, an intermediate peak in the overlap ratio is observed between 5 to 10 hours in Figure 4(d). This peak coincides with the period of exponential growth at the beginning of the simulation, where the rapid addition of new cells leads to a temporary increase in overlaps. As the growth rate slows due to substrate depletion, the overlap resolution becomes more manageable, resulting in a decrease in the overlap ratio.

Comment 2.8: Please signal in Figure 3(a) the cell overlaps that you mention in the text.

Reply 2.8: The figure has been edited to mark the overlaps.

Comment 2.9 Please consider putting the names DORA or kd-tree on top of the panels where growth simulations are shown (Figures 3 to 8).

Reply 2.9: The names have been added to the panels.

Comment 2.10: It would be better to reduce the range of the y-axis in figures showing the Overlap Ratio (Figures 3 to 8).

Reply 2.10: The y-axis range has been reduced.

Comment 2.11: In figures showing the Resolution Time (Figures 3 to 8), you write $X10^3$ ms. Isn't this just seconds (s)? Please consider rewriting as seconds.

Reply 2.11: Correct, this has been rewritten as seconds.

Comment 2.12: Please remove the first paragraph in Results and Discussion, which is a loose sentence (a copy from within paragraph 2).

Reply 2.12: The paragraph has been removed.

Comment 2.13: In Materials and Methods, first paragraph, Figure 3(a) is cited instead of Figure 1(a).

Reply 2.13: This has been corrected.

Comment 2.14 Please check typos, e.g., "Inidividual," "to produce the replicate," "g/L," etc.

Reply 2.14: Typos have been corrected. Thank you for the helpful remarks.

References

Bert Wang-Chak Chan. Lenia-biology of artificial life. *arXiv preprint arXiv:1812.05433*, 2018.

Kazuya Horibe, Keisuke Suzuki, Takato Horii, and Hiroshi Ishiguro. Exploring the adaptive behaviors of particle lenia: A perturbation-response analysis for computational agency. In *Artificial Life Conference Proceedings 35*, volume 2023, page 40. MIT Press One Rogers Street, Cambridge, MA 02142-1209, USA journals-info â€¦, 2023.

Sanyam Jain, Aarati Shrestha, and Stefano Nichele. Capturing emerging complexity in lenia. In *Italian Workshop on Artificial Life and Evolutionary Computation*, pages 41–53. Springer, 2023.

Zhiwei Ji, Ke Yan, Wenyang Li, Haigen Hu, and Xiaoliang Zhu. Mathematical and computational modeling in complex biological systems. *BioMed research international*, 2017(1):5958321, 2017.

Jialiang Wu and Eberhard Voit. Integrative biological systems modeling: challenges and opportunities. *Frontiers of Computer Science in China*, 3:92–100, 2009.
