# Supplementary material for: A Discretized Overlap Resolution Algorithm (DORA) for resolving spatial overlaps in individual-based models of microbes
Source: PLoS Comput Biol. 2025 Apr 21;21(4):e1012974. doi: 10.1371/journal.pcbi.1012974 (PMC12124742; doi:10.1371/journal.pcbi.1012974)
Supplement: Attachment — Submitted filename: PLOS Computational Biology(Review).docx [file pcbi.1012974.s004.docx]

PLOS Computational Biology

A Discretized Overlap Resolution Algorithm (DORA) for Resolving Spatial Overlaps in Individual-based Models of Microbes

**Summary**

The manuscript introduces the Discretized Overlap Resolution Algorithm (DORA) aimed at resolving cellular overlaps in individual-based models (IBMs) of microbial growth. DORA uses a grid-based framework to manage spatial overlaps, reducing computational costs compared to traditional pairwise comparison methods. The authors evaluate DORA's performance in simulating microbial colonies and biofilms under different nutrient conditions, demonstrating its computational efficiency and accuracy.

**Major Comments**

The manuscript primarily focuses on a deterministic approach. Consideration of stochastic elements, which are often relevant in microbial modeling, would broaden the applicability of the algorithm. The current approach is deterministic. How would the algorithm adapt to stochastic IBMs, where random elements influence cell behavior and interactions? The algorithm handles horizontal and vertical movements. Diagonal movements should also be considered to fully capture the spatial dynamics of microbial growth.

The manuscript lacks a comparative analysis with other off-lattice agent-based modeling techniques or the particle Lenia approach. These methods allow for arbitrary ODEs to adjust particle overlap, potentially offering more flexibility. Highlighting the advantages and limitations of DORA relative to these approaches would strengthen the manuscript. A detailed discussion of the superiorities of DORA compared to other existing methods (other than kd-tree) would be beneficial. This includes scenarios where DORA excels and where it might be limited.

The description of the algorithm is clear but could benefit from more illustrative examples or pseudocode to guide readers through the steps, especially those less familiar with computational modeling.

**Minor Comments**

On page 2, the manuscript should refer to Figure 1, not Figure 3. Please ensure accurate figure references.

Ensure consistent use of terminology throughout the manuscript. For example, the terms "cell" and "bacterial cell" are used interchangeably; choose one term for consistency.
